# SUPPLEMENTING GLYNAC IN AGING IMPROVES GLUTATHIONE, MITOCHONDRIA, AND AGING HALLMARKS: A RANDOMIZED CLINICAL TRIAL

**DOI:** 10.1093/geroni/igac059.389

**Published:** 2022-12-20

**Authors:** Rajagopal Sekhar, Premranjan Kumar, Chun Liu, James Suliburk, Jean Hsu, Farook Jahoor, Charles Minard, George Taffet

**Affiliations:** Baylor College of Medicine, Houston, Texas, United States; Baylor College of Medicine, Houston, Texas, United States; Baylor College of Medicine, Houston, Texas, United States; Baylor College of Medicine, Houston, Texas, United States; Baylor College of Medicine, Houston, Texas, United States; Baylor College of Medicine, Houston, Texas, United States; Baylor College of Medicine, Houston, Texas, United States; Baylor College of Medicine, Houston, Texas, United States

## Abstract

Oxidative stress (OxS), mitochondrial dysfunction and aging hallmarks are important contributors to aging, but effective solutions to correct these defects in older adults (OA) are lacking. Via earlier translational studies we discovered that supplementation of GlyNAC (combination of glycine and N-acetylcysteine) improves/corrects these defects. We conducted a double-blind, placebo-controlled (RCT) in 24 OA (mean age 71y) to definitively determine the effects of supplementing GlyNAC vs. isonitrogenous placebo (alanine) for 16-weeks on intracellular glutathione (GSH), OxS mitochondrial function, inflammation, insulin-resistance, endothelial function, physical function, body composition and multiple aging hallmarks. 12 YA (mean age 25y) served as young controls and received GlyNAC for 2-weeks. Subjects were studied before receiving supplementation study, and after receiving supplementation for 2-weeks (OA, YA) and 16-weeks (OA). The RCT found that compared to YA, the OA had severe GSH deficiency (red-cells, muscle), mitochondrial dysfunction, OxS (TBARS, F2-isoprostanes), diminished physical function (gait-speed, muscle strength, exercise capacity), elevated waist-circumference and systolic blood pressure, and multiple hallmarks defects of aging (affecting mitochondrial function, mitophagy, nutrient sensing, inflammation, insulin-resistance, genotoxicity, stem-cells and cellular senescence). GlyNAC supplementation for 2-weeks rapidly improved several defects, and further improved/corrected multiple defects after 16-weeks. No improvements were seen in YA receiving GlyNAC, or in OA receiving the alanine placebo, suggesting that protein supplementation per se in OA does not improve defects. The results of this RCT provides proof-of-concept that GlyNAC supplementation improves/reverses GSH deficiency, mitochondrial dysfunction, OxS, inflammation, physical function/strength and multiple aging hallmarks. GlyNAC could be a novel, simple and safe nutritional supplement to improve/reverse age-associated defects and promote health in aging humans.

